# Identifying Healthcare Professional Roles in Developing Palliative Care: A Mixed Method

**DOI:** 10.3389/fpubh.2021.615111

**Published:** 2021-03-03

**Authors:** Wadi B. Alonazi

**Affiliations:** King Saud University, Health Administration Department, Riyadh, Saudi Arabia

**Keywords:** palliative care, mobilizing healthcare, quality of care, institute of medicine, health governance

## Abstract

**Background:** Creating a holistic approach in healthcare services is the ultimate aim for the integrated healthcare system. Theoretically, healthcare policy makers constantly expected optimal operations within the hospitals through capitalizing the maximum potential of healthcare expertise, professionals, practitioners, and supporting staff. The objective of this study is to explore the role of healthcare individuals to sustain effective palliative care programs in a safe environment with high-quality of care.

**Methods:** This study employed a mixed method (qualitative and quantitative) to accomplish the set objective. For this purpose, a balanced sampling technique was adopted and 28 healthcare professionals were selected in two stages (last week of January and the 1st week of February, 2020). These respondents were playing significant role in palliative care policy making process. In the first stage, respondents were classified into three parallel groups to document the major factors affecting palliative care reforms. To minimize the chance of individual biases, each group was supervised by an independent healthcare professional who was not involved in the study. Then, in the second stage, respondents were divided into two clusters for further abstraction of themes to analysis the data. In this phase, each group was comprised on 14 individuals. Data were transcribed, coded, and analyzed (subjectively and objectively) by using NVivo 12 to extract the final themes. These themes were described and analyzed quantitatively for further catchphrases abstraction to identify significant components.

**Findings:** The initial results incorporated 36 key factors in building effective and sustained palliative healthcare centers. The domains were feasible and practical as they homogeneously patterned within cultural change. These were quality of care, effective management, institute of medicine criteria, and health governance. The Spearman correlation matrix showed significant relationships between the four critical components (*P* < 0.01 and *P* < 0.05).

**Conclusions:** This study explored and identified the significant factors that healthcare professional might consider to make their role more productive and effective in palliative care centers. The key findings also indicated the need of comprehensive periodic assessment especially from the perspective of managerial implications and quality of care.

## Introduction

Most commonly, the prevailing purpose of a palliative care (PC) agenda is to support patients and their families in overall improving their quality of life, including physical, mental, social, spiritual and psychological well-being when they undergo extreme-critical illnesses. Certainly, PC comforts in the early diagnosis, prognosis of pain or any other spiritual symptoms of patients through precise and continuous medical assessment. This comfort level might be provided by the competent healthcare professionals who treat patients with high quality of care in PC (1). This services is provided with respect, modesty, and full support of medical services to those people living with a serious illness (2). Globally, there are many controversies inter alia in the key groups of stakeholders demand to make the PC as an integral part of healthcare service providers. However, most of the PC advanced economies are still ineffectively integrated in their standard clinical practices (3). It is quite evident in many emerging healthcare systems, there is a lack of awareness about the intensity of PC services and their benefits to the society and the significance of underutilized rare resources at large. Currently, it may become essential for healthcare providers, professionals, and practitioners to realize the economic consequences and burden of PC in healthcare sector specifically in emerging economies.

The World Health Organization (WHO) survey indicated that almost one among eight people of the global population age group was projected to be equal or more than 65 years old. More than 75 million people will be living in dementia by 2030 (4). Based on this prognostication, it might be contended this number could increase significantly in the next few decades especially when we closely consider the total population age group to be equal or more than 85-years. Thus, it is postulated that there will be obvious needs of PC educational programs, skill, and knowledge (5). Globally, seriously ill hospitalized patients became a real challenge for healthcare service providers, whether to compromise or not on the quality of their lives in the domain of PC (6). As highlighted in various studies a number of initiatives have been taken by healthcare providers in terms of advanced technology and infrastructure for sustainable medical services in the domain of PC, but still there is a need to improve human related services (7–9). These are heavily based on personal activity with practical collective-mindsets to introduce new reform systems, strengthening healthcare related outcomes, and enforcing healthcare system users in general, particularly healthcare professionals (10–12).

In an extensive review of the relevant literature in the domain of PC, healthcare professionals' roles may not be well-explored seriously, specifically in emerging economies in respect of reforms and quality of care among older adults.

For instance, the activities of healthcare experts are still limited to their assigned tasks. Their involvement is quite little in the process of policy and decision making; consequently, they are unable to go above and beyond their normal job responsibilities to make effective efforts.

Baxter and his associates concluded that healthcare professionals' roles were significant to implement new reforms and obtain funding from the concerning bodies to improve the overall quality of healthcare systems especially in PC (13–15). There is dire need to systematically address PC issues including new reforms and to apply evidence based practices in public healthcare systems around the globe (16, 17). Though, healthcare operations are complex in its own nature, policy formulation and decision making processes are the true reflection of healthcare systems outcomes (18–20).

In line with these points of view, Ministry of Health (MoH) in Saudi Arabia endeavors to monitor, finance, and manage a multifaceted healthcare system where more than 470 hospitals, 2,325 primary healthcare centers, as well as many private and semi-governmental agencies provide advanced healthcare services for more than 30 million people (21, 22). Nevertheless, in real practices within the domain of medical services such as PC is not well-recognized among many practitioners and professional (23). A number of theorists and researchers in the context of PC argue that there is a lack to measure the organizational performance based on the perspective of individuals involved in the hospital operations (24, 25).

The findings of various studies indicated that healthcare initiatives required capacity building in the areas of planning, budgeting, and management performance for the sake of empowering stewardship process (26, 27).

More than 85% of healthcare professionals in public, semi-governmental and teaching hospitals work under the authority of the Saudi national health system (SNHS) (28). Such high percentage employed by SNHS raises a fundamental question concerning their role in delivering the PC services in adult care centers in relations to healthcare governance (29). Ideally, effective healthcare governance may have a responsiveness, accountability, and transparency to design and implement effective protocols in different health levels. Effective healthcare system depends on the integrated acts of the individuals, governing bodies, and the participation of stakeholders, as identified by Caroline et al. (30).

A comprehensive analysis of government policies seem to be widely implied to improve the process of PC; but still there is a lack of national professionals and experts and heavily depend on foreign workforce in the SNHS. This deficiency contributes ultimately to develop barriers to implement in true spirit the given protocols. Hence, professionals might be unable to play their significant roles in building up the sound institutional performance. Generally, PC services in Saudi Arabia is limited to the urban cities and within tertiary healthcare services. However, rural areas may have a lack of some essential medical services including PC services (31). An explanatory and descriptive study design was employed to find out the healthcare professionals' role to mobilize the PC services and effectively utilization of rare resources.

## Materials and Methods

In early 2020, a workshop had been held, with the primary objective of initiating a strategic plan for public health in the SNHS from different perspectives. More than 72 of multidisciplinary healthcare professionals were invited to participate in this workshop. The target population of the present study was all the participants who attended the workshop. Initially, three focus-group discussions were arranged based on their specialty. Only 28 health professionals, as their roles were also to formulate policies in the SNHS, agreed to participate in the two stages of the study. In the first phase of the study, the participants were categorized into three groups: 8 physicians; 8 medical engineers; and 12 healthcare professionals. The aim was to identify the critical factors from the perspectives of these professionals to provide quality of PC services in urban and remote areas.

In the second phase of the study, the participants were categorized into two clusters for focus group discussion. The purpose of this discussion was to comprehend participants' perspectives in subjective manners for the significance of professionals' roles to effectively mobilize PC services. During the focus group discussion, a number of themes were extracted to ensure the subjectivity of the quality of PC services. The tally matrix was used between both phases of the study to ensure the consistency of critical factors and themes. The scientific protocols were followed to ensure the participants' confidentiality and ethical approval was obtained from the relevant authorities.

### Procedures

With the support of the MoH staff, the principal investigator started with the initial training activities with healthcare professionals including physicians, engineers, and administrators. Training included the definition of PC reform, the objective of the study, liaise roles, how to derive key factors, and the constancy between policies issued by MoH and healthcare activities within the capacity of the study participants. Throughout this process, many healthcare professionals showed that they had acquired the necessary training and experience.

### Subject and Setting

MoH holds periodic workshops to facilitate the role of healthcare professionals in achieving health reforms in Saudi Arabia. In the 1st day, the principal investigator introduced the research objectives to his own team members, and explicitly explained to them the participation of study subjects was vital and participants' identity along with data will be kept confidential. It was mandatory that the interested participants have to sign the consent form. Next day, participants were divided depending on their occupations and one leader was assigned to each group. In every stage of the study, the participants spent almost 2 h addressing the key-factors to mobilize healthcare reforms specifically quality of PC services.

### Data Collection and Analysis

The collected data were based on unanimously agreed factors among all study participant and their discussion was recorded for thematically analysis. Criterion validity was used to compare the obtained data of each focus group against the facilitators' noted documents. Thematic data saturated until there were no new emerging ideas in the data from the two groups. The facilitator used a guide to explain the purpose of each group, review the focus group rules and other information that was essential to the participants. The script helped to enhance reliability in high-focus group.

## Results

The following table summarizes the socio-demographic characteristics of the participants (Table 1).

**Table 1:** Demographic characteristics of the sample.

**Characteristics**	**Male**	**Female**	***n* = 28**	**%**
Physicians (PHs)	3	5	8	28.6
Engineers (ENs)	6	2	8	28.6
Health Administrators (HAs)	7	5	12	42.9
**Education**				
PhD holder	2	1	3	10.7
Masters' degree holder	4	2	6	21.4
Bachelors' degree holder	10	9	19	67.9
**Experience**				
>10 years of experience	10	7	17	60.7
5 ≥ 10 years of experience	5	3	8	28.6
<5 years of experience	1	2	3	10.7

The focus groups identified the magnitude of health reforms, as well as the concept of access to PC, as shown in Table 2. The results displayed in Table 2 reported the challenges and expected benefits of healthcare system reforms and PC services.

**Table 2:** The expected benefits of the new reforms and PC services.

**Statement**	**Category**	**Nationally**			**Regionally**			**Provider-based**		
		**M**	**F**	**%**	**M**	**F**	**%**	**M**	**F**	**%**
The PC reform	PHs	1	2	38	1	2	38	1	1	25
should be:	ENs	3	0	38	1	0	13	2	2	50
	HAs	3	1	33	1	2	25	3	2	41.7

When healthcare professionals were requested to indicate whether the new reforms may increase access to PC services, the majority of healthcare professionals agreed that such reform would lead to better access, as shown in Table 3.

**Table 3:** The expected access to the SNHS after the PC reform.

**The new reform will improve access to PC:**						
**Professionals**	**M**	**F**	**%**	**M**	**F**	**%**
	**Y**	**Y**		**N**	**N**	
PHs	3	3	75	0	2	25
ENs	4	2	75	2	0	25
HA	5	3	66	2	2	44

The thematic analysis of the data shows the elements of effective healthcare system and the quality of PC services (Table 4). Four major themes were explored: quality of care, effective management, implementing the institute of medicine (IOM) recommendations in relation to an effective healthcare system including quality of PC services and sustaining the effective healthcare governance.

**Table 4:** The four major themes were explored based on aggregated consensus of the participants.

**Extracted themes**	**PHs**		**ENs**		**HAs**		**Accumulated rate**
	A^*^	D^*^	**A**	**D**	**A**	**D**	
Quality of care (KI 10)	8	0	8	0	12	0	100%
Effective management (KI 8)	6	2	7	1	10	2	82.1%
Implementing IOM recommendation (KI 12)	4	4	6	2	9	3	67.8%
Maintaining health governance (KI6)	2	6	6	2	7	5	53.5%

* *A, Agree; D, Disagree*.

Table 5 shows the linear relationships between the two focus groups based on the role of healthcare professional, especially in the domain of PC services.

**Table 5:** Spearman's Rho correlation matrix among four themes in the second stage.

**Group**	**Domain**	**Quality**	**Management**	**IOM**	**Governance**
Clusters (*n* = 14)	Quality	1.00			
	Management	0.759^**^	1.00		
	IOM	0.133	0.508^*^	1.00	
	Governance	0.016	0.358	0.328	1.00
Clusters (*n* = 14)	Quality	1.00			
	Management	0.599^**^	1.00		
	IOM	0.458	0.259	1.00	
	Governance	0.328	0.432	0.350	1.00

** *Correlation is significant at the 0.01 level (2-tailed)*,

* *Correlation is significant at the 0.05 level (2-tailed)*.

These results indicated consistency between the two groups. Again, this study analyzed thoroughly certain factors by comparing and interpreting, in depth-patterns, themes, and questions.

## Discussion

This study investigates multiple subtle features practiced by many healthcare professionals that may influence the effectiveness of healthcare reforms and PC services during the transitional period toward government Vision 2030. First, three interrelated healthcare professionals were classified, including physicians, medical engineers, and healthcare administrators. They identified many key issues in the SNHS. The methods used in this paper were scientific and practical in revealing the early stages of effective healthcare system specifically PC services in Saudi Arabia (16, 25, 32, 33).

Commonly, all healthcare professionals stressed on two aspects: financing and regulation of the SNHS. For the effectiveness of healthcare system, PC services may need to utilize the maximum potential of human capital that might be directly involved in the policy and decision making process in MoH. It is evident from the study results, MoH may still need to subsidize the healthcare provisions through stewardship module, but quality should be a priority within PC contexts (34). The key findings of thematic analysis indicated that quality of patients care and PC services were the major concern among many healthcare professionals. The establishment of a safe and effective healthcare system in PC services seriously require the collaboration of many agencies, especially semi government and private healthcare hospitals. The SNHS has already moved into new era of providing effective healthcare system, particularly in specialized care like PC services (35–37). However, the findings of this study highlighted the deep rooted role of MoH activities that creates barriers in the functions of healthcare professionals to deliver their services in expected manners. Obviously, patient-centeredness was not well-constructed within the SNHS (31, 38).

The findings of this study is consistent with many earlier empirical and non-empirical results to indicate strong relationships between management and quality of caregivers in the context of PC services. Nevertheless, in line with the previous studies in the same domain, the findings of this paper indicate the alignment of professional roles are still quite significant to contribute to the overall performance of the healthcare system especially in PC services. For this purpose, SNHS like other developed and emerging economies may need to provide appropriate personnel trainings to meet the expectation of both internal and external stakeholders (19, 20, 39).

Thus, based on the findings of this study, it is argued that in order to improve the overall performance of the healthcare system including PC services, the SNHS may need to develop implementable framework. This model might be based on the theorists, researchers, professionals, policy makers, and practitioners' expertise and their workplace experiences through privatization, partnerships, or stewardship (15). However, the evidence based results of this indicated that the SNHS may also give careful consideration on periodic assessment in the healthcare system to mobilize PC services in Saudi Arabia (40).

### Conclusions

The mixed-methods approach was appropriate to conduct this kind of study and to comprehend the complexity of healthcare systems in the domain of PC services. The key proposition of the present study guided toward this approach to develop evidence based perspective about the reality of healthcare system reforms particularly to mobilize the PC services. The findings of this study established the foundations for the upcoming refinements and new initiatives in the form of Saudi Vision 2030. However, the results also provide bases for theoretical insights and practical technology to theorists, researchers, professionals, practitioners, policy, and decision makers to improve the practices and outcomes in healthcare system for the mobilization of PC services. The respondents of the key proposition of this study have also proposed various ways by which the SNHS may add the excluded principles in its portfolio. The role of healthcare professionals has substantiated to be influential in mobilizing the transformation of the SNHS. All these activities aim at ensuring the full right of dying well as a basic human right.

## Data Availability Statement

The raw data supporting the conclusions of this article will be made available by the authors, without undue reservation.

## Ethics Statement

The studies involving human participants were reviewed and approved by King Saud University (IRB 2019-67-082). Ministry of Health approved the research project on 5 December 2019 (HP 19-127-25). The patients/participants provided their written informed consent to participate in this study.

## Author Contributions

The author confirms being the sole contributor of this work and has approved it for publication.

## Conflict of Interest

The author declares that the research was conducted in the absence of any commercial or financial relationships that could be construed as a potential conflict of interest.
